# Metastatic Niches and the Modulatory Contribution of Mesenchymal Stem Cells and Its Exosomes

**DOI:** 10.3390/ijms20081946

**Published:** 2019-04-20

**Authors:** Matias Valenzuela Alvarez, Luciana M. Gutierrez, Alejandro Correa, Alberto Lazarowski, Marcela F. Bolontrade

**Affiliations:** 1 Remodelative Processes and Cellular Niches Laboratory, Instituto de Medicina Traslacional e Ingeniería Biomédica (IMTIB)—CONICET—Instituto Universitario del Hospital Italiano—Hospital Italiano Buenos Aires (HIBA), C1199ACL Buenos Aires, Argentina; valenzuelamatias25@gmail.com (M.V.A.); lucianagutierrez1987@gmail.com (L.M.G.); 2 Instituto Carlos Chagas Fiocruz/PR, 81350-010 Curitiba, Brazil; alejandro.correa@fiocruz.br; 3 INFIBIOC, Clinical Biochemistry Department, School of Pharmacy and Biochemistry (FFyB), University of Buenos Aires (UBA), C1113AAD Buenos Aires, Argentina; nadiatom@ffyb.uba.ar

**Keywords:** MSCs, exosomes, pre-metastatic niche, metastasis

## Abstract

Mesenchymal stem cells (MSCs) represent an interesting population due to their capacity to release a variety of cytokines, chemokines, and growth factors, and due to their motile nature and homing ability. MSCs can be isolated from different sources, like adipose tissue or bone marrow, and have the capacity to differentiate, both in vivo and in vitro, into adipocytes, chondrocytes, and osteoblasts, making them even more interesting in the regenerative medicine field. Tumor associated stroma has been recognized as a key element in tumor progression, necessary for the biological success of the tumor, and MSCs represent a functionally fundamental part of this associated stroma. Exosomes represent one of the dominant signaling pathways within the tumor microenvironment. Their biology raises high interest, with implications in different biological processes involved in cancer progression, such as the formation of the pre-metastatic niche. This is critical during the metastatic cascade, given that it is the formation of a permissive context that would allow metastatic tumor cells survival within the new environment. In this context, we explored the role of exosomes, particularly MSCs-derived exosomes as direct or indirect modulators. All this points out a possible new tool useful for designing better treatment and detection strategies for metastatic progression, including the management of chemoresistance.

## 1. Introduction to MSCs

The stroma has emerged during the last years as a key regulatory element in normal and pathological tissues. Several populations of cells with well-defined functions, or cell populations with overlapping functions, are able to modulate a given process and finally determine, not only their own, but their neighbor’s cells fate, in an interconnected way.

Within this vision, mesenchymal stem cells (MSCs) are relevant actors in giving origin to cellular stromal elements, with a functional and structural contribution and even determining the tissue architecture. The term MSCs also designates mesenchymal stromal cells, pointing out the heterogeneous nature of this cell population [1]. Initially discovered in the bone marrow (BM), MSCs were later described in practically every tissue, both pre- and postnatal [2]. Main functional characteristics of MSCs are their multipotency and immunomodulatory capacity, among not less important functions, such as their angiogenic ability. All this points to their potentiality for tissue repair strategies and, in general, for niche formation, not only normal niches but also pathological ones, such as an hematopoietic and non-hematopoietic tumor milieu [3,4].

MSCs can give rise to fibroblasts, osteoblasts, chondroblasts, adipoblasts, pericytes, and even other cell types. The differentiation trend of each one the cell types will depend on the environment where MSCs reside. Thus, several signals would determine a percentage of differentiation into different cell types. For example, bone marrow MSCs (BM-MSCs) would give rise predominantly to bone and cartilage, while adipose tissue MSCs (AT-MSCs) will originate predominantly adipocytes. However, isolated from its surroundings and in culture conditions, these cells will differentiate almost in equal proportions to the three main cell lineages (bone, cartilage, and fat). In these conditions, AT-MSCs will show a more pronounced tendency to become adipocytes while BM-MSCs will be more prone to become osteocytes and chondrocytes, due to epigenetic memory acquired at the tissue where they were educated [5,6,7].

MSCs are the “mothers” of fibroblasts, which are highly motile cells that come to the rescue of any given tissue when an injury arises [8]. For instance, the formation of a scar is a result of collagen deposition from the fibroblasts that arrived into a transient matrix. Like mother like son, MSCs are highly motile cells with the ability to move within their tissue of residence, from there to adjacent tissues and even to distant tissues, with a notable homing capacity. That is the case of BM-MSCs or AT-MSCs arriving into a growing tumor or into tissues under stress [9]. In this context, a tumor stroma would build-up, in a biochemical way, a microenvironment that will resemble that of a damaged tissue [10,11]. MSCs have been considered as a cell repository, helping to maintain tissues’ requirements when they are not able to support their own homeostatic demands with their own stem cell populations. In this case, travelling MSCs would home and could help the tissue under stress by becoming a part of the stroma, by recruiting other cells such as immune cells, and/or by secreting exosomes [12,13,14].

To add to the complex scenario, MSCs can suffer mesenchymal to epithelial transition (MET) when exposed to different microenvironmental cues and, under environmental prompt, could go backwards to a mesenchymal phenotype suffering epithelial to mesenchymal transition (EMT). Rubio et al. showed that transformed MSCs could give rise to primitive carcinomas through MET [15]. A process of dedifferentiation associated with EMT, that promotes stem-like properties, has been associated with the formation of fusion hybrids between MSCs and lung cancer cells [16]. In addition, MSCs were shown to induce MET in tumor cells, thus increasing cell migration, plasticity, and other tumor progression properties [17,18]. Cancer associated fibroblasts (CAFs), which are activated fibroblasts, could originate from recruited stromal cells, like MSCs, or from resident fibroblasts and add protumorigenic properties to a general permissive stroma [19]. Additionally, endothelial cells (ECs) can turn into fibroblastic cells through EMT, originating CAFs and indicating that not only mesenchymal-like cells in the stroma are able to contribute to a highly remodeling phenotype [20].

Therefore, a stroma arises as a synergistic interconnected function rather than as a sum of the functions of its individual parts, with any action on any cell exerting a modulation in the environment, which in turn would modify back the cells in a circuit-like form. To add to the complexity, other signaling mechanisms are implied in the communication between cells. Exosomes are also involved in cell to cell signaling, transferring molecules via a system that involves trafficking between membrane vesicles [21].

## 2. Tropism of MSCs

Cell migration in physiological conditions and in non-embryological tissues is associated with immune cells, fibroblasts, in some measure to endothelial cells, and to stem cells. In disease settings, tumor cells may present independent migration capacity [22].

As mentioned, MSCs are highly motile cells with intrinsic tropism ability, which is not exclusive of a type of organ or a pathological condition, but it is associated with microenvironments enriched in growth factors (GFs), cytokines, and chemokines. Inflammatory environments are particularly rich in these factors, thus recruiting MSCs through a variety of receptors. Receptors, such as CXC chemokine receptors (CXCR) 4, 5, 6, CXCR1, CC chemokine receptors (CCR)1, 4, 9, 10, and c-met have been associated with tumor tropism [23]. In general, these receptors and others are also implied in immune cell recruitment and establishing chemotactic axis responsible for MSCs directed migration.

A chemotactic gradient is thus generated and guides MSCs towards a target site [24]. MSCs arriving to a given environment are educated in that new niche and, thus, their functional responses would be modulated. Hypoxia can contribute to MSCs-directed migration [25]. Regarding the plethora of GFs that MSCs can find in a new niche, López Ponte et al. have analyzed the in vitro migration capacity of human BM-derived MSCs, preincubated or not, with IL1-β and TNF-α and their response to a variety of GFs and chemokines. They showed that the preconditioning with inflammatory cytokines affected these cells migratory response, since preincubation with TNF-α increased MSCs migration toward chemokines, while their migration toward most of the evaluated GFs was not altered by the preconditioning. Furthermore, TNF-α increased the expression of CCR2, 3, and 4, with all of these suggesting that local or even systemic environments would influence MSCs’ final homing and, probably, their future functional responses in a new environment [26].

For any cell to arrive into a given target site, it must cross the endothelial barrier. The mechanisms associated with extravasation have been described for different cell types. Immune cells have provided the first and the paradigmatic model [27], which have been associated with extravasation for other kind of cells, such as tumor cells, and are also described in MSCs [28]. More recently, an alternative extravasation mechanism for MSCs was described, termed angiopellosis. It consists of a multi-step process with an active participation of ECs, where circulating MSCs attach to ECs, and these in turn cover attached MSCs with cell membrane projections, eliciting their displacement into the target tissue. This requires a longer time than leukocyte extravasation [29].

MSCs have been considered as depot cells that may arrive into a tissue under physiological or pathological remodeling. The ability to migrate is a stemness-associated characteristic, which is evidenced during embryogenesis but is retained during adult life [30]. Circulating cells with stem cell or progenitor characteristics have been reported since the 1960s [3,31,32]. Decades later, bone marrow-derived cell populations were shown to migrate and home into injured tissues. Examples include CD34+ and FlK1+ cells [33,34]. MSCs have been labeled to allow tracking after local or systemic administration during biodistribution studies. The patterns of MSC distribution differ in tissue-damaged animals or uninjured animals. Thus, in disease-free animals MSCs were tracked into the lungs, spleen, and liver, while MSCs showed a preferential location in damaged sites in animals with injured tissues [8,35]. MSCs were shown to home into tumors in a variety of models [4,36,37,38]. Tumors undergo a high remodeling activity and determine an inflammatory milieu, in this context they constitute a preferential site for specific MSC migration and final location [10].

It is evident that MSCs play a key role in tissue homeostasis and regeneration through an active secretive activity. Additionally, they serve as a cell niche for other cell types, favoring cell expansion and survival, and even neuroprotective action through the secretion of neurotrophic and anti-inflammatory factors [39]. In addition to promoting MET in tumor cells [17,18], favoring properties that stimulate a metastatic behavior, MSCs could have an active role in creating a metastatic niche and establishing a secondary tumor growth site, for example by facilitating the entrance of tumor cells through the endothelial barrier [40,41] or by homing into a secondary site and, from there, actively recruiting tumor cells (unpublish results from our laboratory).

## 3. Is It a Matter of Quantity or a Matter of Quality?

Through experimental evidence we know that all infused MSCs do not reach a given target site, but they are able to induce functional responses anyway, conducting to expected clinical benefits [42]. Therefore, the following question arises: Why can a few cells achieve a great deal of response?

Any potential therapeutic use for MSCs would depend on their ability to migrate and home into a target site. Depending on the administration route, for example local or systemic, the cell cargo entering the target would vary [42]. Tracking studies, analyzing biodistribution of infused MSCs, have suggested that the quantity of infiltrating MSCs may not be sufficient to provide a substantial contribution to the targeted site, thus leaving the increasingly robust idea of a paracrine effect of MSCs, given the functional effects observed in different models [43,44,45]. Interestingly, in a rat model, the paracrine action of MSCs, in the form of exosomes, was shown to exert a therapeutic effect by enhancing wound healing through collagen synthesis and angiogenesis [46]. Cells’ secretion is not composed only of molecules such as GFs, cytokines, chemokines, etc., but also of extracellular vesicles (EVs) [47]. The molecular packaging of these vesicles is not random, but highly enriched in specific molecules, including proteins, lipids, and different types of RNAs, pointing at the specificity of action. Furthermore, extracellular vesicles from different sources have conserved proteins [21,48]. Currently, the paradigm based on a functional effect of MSCs, in the evidence of a few amount of cells reaching a target site, rests upon the secreted fraction, including the classical effectors, such as GFs, chemokines, and EVs, among others [49].

Information related to the difference in an EVs cargo in different conditions has been growing. MSCs release EVs upon physiological and/or activating signals or under stressor signals, so the environmental cues impact in the MSCs phenotype and, as a consequence, they modify their behavior. This could happen by modifying the secretome of MSCs including modification of an EV’s content. So, in terms of quality, EVs released by MSCs cultured under hypoxia are not the same as those released by MSCs cultured under normoxia, mainly by differences in their content, such as miR-21-5p, which is significantly upregulated in EVs from hypoxic MSCs. This could promote lung cancer development by reducing apoptosis and promoting macrophage M2 polarization [50]. Another important aspect to take into account is that MSCs not necessarily need to the leave the resident tissue, given that they have the ability to modify the cargo of EVs upon different signals, allowing them to modify or modulate different target tissues without leaving their residence tissue. These could be related to the role of exosomes in the determination of cell fate during normal development [51]. 

## 4. The Matter of “How”: The Existence of Exosomes

Initially it was believed that EVs sprouted directly from the plasmatic membrane (PM), but in the 1980s it was proven that little vesicles were created inside of an endosome, which lead to the formation of a multivesicular body (MVB) that could fuse with the PM and release the vesicles that were contained in the MVB [52,53]. In 1987 these vesicles with endosomal origins were called “exosomes” [54]. The existence of these exosomes secretion pathways was confirmed in a multiplicity of models, such as epithelial and tumor cells [55,56].

EVs could be classified into three main classes based on their biogenesis, exosomes, microvesicles, and apoptotic bodies. Exosomes are of endocytic origin and are the smallest among the EVs (30–150 nm diameter), microvesicles or ectosomes are large vesicles (100–1000 nm diameter) that directly sprouted from the PM, and apoptotic bodies are those vesicles released during the apoptotic process (50–5000 nm diameter) [57,58,59]. The International Society for Extracellular Vesicles (ISEV) released minimum experimental requirements for the correct characterization and definition of EVs and their function in 2014, and updated these requirements in 2018, as follows: (1) Consider the use of terms for EVs that refer to a physical (size or density) or a biochemical characteristic (biochemical composition) or based on a description of conditions or the cell of origin; (2) EVs should be isolated from extracellular fluids, that is, from conditioned cell culture mediums or body fluids; (3) semi-quantitative proteomic characterization with enrichment in the transmembrane and cytosolic proteins with a membrane-binding capacity; and (4) characterization of single vesicles within a mixture should be performed to provide an indication of the heterogeneity of the isolated EVs [60,61].

Exosomes transfer information to the target cells through three main ways, as follows: (1) Receptor-ligand interaction; (2) direct fusion with PM, and (3) endocytosis by phagocytosis. The mechanisms involved in each possible pathway are not fully understood but it is well documented that phagocytic cells have a greater uptake of exosomes than non-phagocytic cells. The uptake of exosomes by the recipient cell is energy dependent and low pH facilities this process [62,63,64,65,66,67,68]. EVs are different from other cell signaling pathways based on their capacity to transport different kinds of molecules. This means that their signaling capacity is greater than common cell signaling mechanisms, such as hormones, or soluble factors, like GFs or cytokines. This is why EVs (exosomes included) have been suggested to be complex extracellular organelles mediating intercellular communication, giving birth to the term communicasomes [68].

These nano sized vesicles, of endocytic origin, are composed of and contain a varied composition of macromolecules, including proteins, lipids, mRNAs, and miRNAs. The discovery and analysis of the proteins that are contained within exosomes indicate that protein composition is highly dependent on the cell that formed the exosomes, meaning that they contain similar proteins to the cell they originated from [69]. Studies done in different cells, such as B lymphocytes [70], dendritic cells [71], and intestinal epithelial cells [55] show that there are common, as well as cell-type specific, proteins within exosomes. Common proteins among exosomes are the Rab proteins, which could participate in the docking and fusion with the PM of the recipient cells [72]. Annexins, adhesion molecules, apoptosis proteins, heat shock proteins, and tetraspanins (CD9, CD63, CD81, and CD82) [55,71,73,74] are classically found within exosomes of different origins. The first proteomic studies of exosomes demonstrated that they represent an specific subcellular compartment, given that the content corresponds to endosomal, PM, and cytoplasmic proteins and they did not find nuclear, mitochondrial, or Golgi proteins [74]. Even though exosomes pose the enrichment of endosomal proteins, in comparison to EVs born from PM, the proteomic profile of these two kind of vesicles largely overlaps [75].

Apart from proteins, other important macromolecules composing exosomes are lipids, like cholesterol, phospholipids, diglycerides, sphingolipids, and glycerophospholipids. The ratio of lipids found in exosomes is up to four times greater, when compared to the parental cell, and this may account for the increased rigidity of the membrane of the exosomes [72,76]. Besides lipids, some specific bioactive lipids are found inside exosomes like prostaglandins and leukotrienes [77,78]. Another type of biomolecule found in exosomes are nucleic acids, such as DNA (dsDNA, ssDNA, and mtDNA) [79,80] and different types of RNAs (like mRNA, lncRNA, and miRNAs) [73,81,82]. The most common mRNA, miRNAs, proteins, and lipids found in exosomes have been deposited in ExoCarta (www.exocarta.com), Vesiclepedia (www.microvesicles.org), and EVpedia (www.evpedia.info).

The exosomal process is an orchestrated and deliberated activity (formation, content selection, loading, trafficking, and released). A few years ago an in vitro study determined that tumor cells have a 10-times higher exosome release rate than normal cells and these finding disproved the most commonly accepted theory of passive exosome sorting and loading [83]. The aberrant exosomal process in cancer cells can be partially explained by cancer increased endocytosis and the over-expression and recycling of different surface receptors, such as ABC transporters, GF receptors, and various importers [84,85,86,87]. The exosomal content varies between physiological and pathological conditions and original cell types. The difference between exosomes released from a healthy tissue (normal cell) and those released from cancer cells has been extensively investigated in various studies, indicating that the rate of exosomal release and their content (mainly miRNAs) is increased and different. It has been demonstrated that the exosomes released from cancer cells, unlike the ones released from normal cells, are enriched with miRNAs that are associated with the RISC loading complex. The RISC complex is essential for the maturation of miRNAs, which gives them the capacity to silence target genes [88,89].

## 5. A Role for MSCs in Niche-Related Tumor Environments: MSCs, Exosomes, or Both?

A tumor is made-up of two main populations of cells, tumor cells *per se* and “non-transformed” cells. The interactions established between them are mediated by cytokines, chemokines, GFs, inflammation related factors, and other cell to cell communication mechanisms involving EVs [90,91,92,93,94,95,96]. All these cell populations and the interactions between them define the tumor microenvironment (TME). In the context of TME, the interaction between MSCs (representing the associated tumor stroma) and tumor cells is established through different soluble signals released by both cells types and by paracrine signaling mediated by EVs. Exosomes released by the mass of tumor cells and by the associated tumor stroma promote different biological processes, such as proliferation, resistance to apoptosis, and angiogenesis, and are capable of enhancing the systemic entry and progression of cancer cells along the metastatic cascade [84,97,98]. This highlights the importance of understanding exosome biology involved in the progression of metastatic disease.

Metastasis is a multi-step process, where some of the cells of the primary tumor acquire migratory capacity associated with a change of phenotype, termed EMT, which allows them to disseminate from the primary tumor site to distant target tissues. Besides its biological complexity, the metastatic process presents itself as a major clinical challenge, given that 90% of the mortality of patients diagnosed with cancer is attributed to the presence of metastasis in distant organs [99,100]. In 1889, Paget proposed his theory of “seed and soil”, where he stated that metastasis occurs in an organ-specific manner, depending the cancer type [101], and this concept of metastasis growth specificity has been validated clinically and experimentally in different models [102], showing that cancer cells can be found circulating through different organs, but only selective sites consistently develop metastatic tumor deposits [103]. Presently, it is widely accepted that the spread of cancer cells to secondary organs is indeed promoted by the prior formation of a specialized environment at distant sites, termed the pre-metastatic niche.

The pre-metastatic niche is constituted by the formation of a permissive environment that allows the implantation of metastatic cells and creates a suitable context for the selection of the cells that will be able to survive and thrive in this new soil. Paget gave the first clues regarding the tropism of primary tumors for secondary metastatic sites [101] and it is believed that exosomes contribute in these processes directly and indirectly. They could directly modulate the future metastatic tissue and start the formation of a pre-metastatic niche by modification of the local conditions, such as cell population, irrigation, or nutrient supply, and could indirectly influence the formation of this permissive milieu by preconditioning BM-derived cells, such as MSCs, to migrate to the target tissue and start preparing the parenchyma for the cancer cells [9].

The first approaches to studying pre-metastatic niche formation have shown that VEGFR-1^+^BM-derived cells (BMDCs) accumulate at pre-metastatic sites in organs different to the site of the primary tumor and before the arrival of any cancer cells [104]. These cells, and the abundant fibronectin present in the parenchyma of the pre-metastatic niche, represent an attractive docking site for the disseminating tumor cells. The mobilization of BMDCs from the BM and their recruitment to the future metastasis site was thought to result from VEGF and placental GF (PGF/ PlGF) secreted by the primary tumor [104]. Other inflammation related factors, such as VEGF-A, TGF-β, and TNF-α, released by the primary tumor, have also been reported to induce the recruitment of BMDCs to the formation site of the pre-metastatic niche and these inflammation chemokines also induced the expression of inflammation proteins (S100A8 and S100A9) that made the parenchyma strongly chemoattractive for BMDCs and tumor cells [105]. Local tissue remodeling is essential for the colonization of the metastatic site and that is why matrix metalloproteinases (MMPs) are also upregulated at the pre-metastatic niche, specially MMP9, which can act upon facilitating tumor cell invasion and releasing GFs and chemokines retained in the extracellular matrix [104]. It is hypothesized that BMDCs recruited into the pre-metastatic niche have the function of creating an immune sanctuary where metastatic cells are able to survive and proliferate without detection [106].

The tumor-secreted factors responsible for the priming of the “soil” are diverse cytokines, GFs, chemotactic factors, and extracellular matrix remodeling enzymes [104,105,106,107,108], but more recently exosomes have been proposed as an integral part of complex tumor-host interactions, particularly during pre-metastatic niche establishment and maintenance [109,110]. This concept can be included in Paget’s theory, where the pre-metastatic niche is the “soil”, the tumor cells would be the “seeds”, and exosomes would act as “fertilizers” facilitating the establishment of seeds in the new soil. Supporting this hypothesis, Hoshino et al. [111] described the role of exosomes integrins (ITGs) in the determination of organotropism, thereby mediating nonrandom metastatic patterns.

Inflammation is a driven force for tumor development and is one of the basic factors for the formation of a pre-metastatic niche. Exosomes contain different ITGs that can modulate the expression of different proinflammatory elements of S100 family within the pre-metastatic microenvironment [110]. Another important factor is the immune suppression in the pre-metastatic niche and it has been proven that exosomes can carry programmed death ligand 1 (PD-L1), that can bind to programmed death receptor 1(PD-1) found in macrophages, T and B cells inhibiting the activation and proliferation and inducing apoptosis, promoting the formation of a suitable immune environment [112]. Tumor exosomes act synergistically with the inflammatory cytokines produced by local proinflammatory microenvironments in the recruitment of suppressive immune cells, such as tumor associated macrophages (TAMs), tumor associated neutrophils (TANs), and regulatory T cells (Treg) [113], to the pre-metastatic niche, supporting its formation. It is important to remark that, like the heterogeneous nature of cancer cells, exosomes also are heterogeneous and this could be reflected in the negative effects of exosomes in some experimental models, were the formation of a pre-metastatic niche was inhibited by favoring immune surveillance [114].

Exosomes derived from MSCs can contain a wide variety of cargo molecules and factors, which is why they have been in the spotlight for the last couple of years in the field of regenerative medicine [115]. MSCs-derived exosomes can support tumor growth in vivo, increase migration, participate in the acquisition of apoptosis resistance, and stimulate angiogenesis, promoting the formation of a pre-metastatic vascular microenvironment [98,116,117]. New blood vessels in the pre-metastatic context are the carriers of not only EVs and different cells, but could also bring circulating cells to the future metastasis site. Another important modification of blood vessels is the induction of vascular permeability, which facilitates the extravasation of cells into the pre-metastatic site. Some miRNAs (miR-105, miR-25-3p) have been suggested to be involved in the vascular permeability and leakage [118,119]. As mentioned above, MSCs have the ability to release a wide range of cytokines and GF in the tumor environment and these molecules impact diverse biological processes like proliferation, apoptosis, chemoresistance, angiogenesis, and metastatic progression. Multiple evidence supports our group’s idea that positions MSCs as a key stromal component of the tumor microenvironment, with them being of such importance that tumor cells would release different factors and exosomes that would allow MSCs to adopt a chemoresistance phenotype with an increase in pluripotency markers, enabling them to survive even in adverse conditions and keeping on the release of molecules to ensure the cancer cells’ survival [120].

Metastasizing cells must acquire suitable functional properties to leave a primary tumor site and arrive into a new niche. An increase in the general motility of tumor cells is an advantage feature to colonize a secondary tumor site. In a breast cancer cell model, Lin et al. demonstrated that exosomes from AT-MSCs activated the Wnt signaling pathway in MCF7 cells, not only increasing their migration, but also their proliferative capacity [14]. Interestingly, exchange of Wnt signals via exosomes was also demonstrated in large B-cell lymphomas, where two distinct tumor cell subpopulations, differing in their stemness state, could use exosomes to communicate as a mechanism that would keep a subpopulation equilibrium within a tumor and may affect tumor progression [121]. Other tumor models are involved in information transference from stromal cells to tumor cells via secreted vesicles. MSCs isolated from gastric cancer were shown to selectively pack into exosome miRNAs able to induce gastric cancer metastatic features through EMT induction in gastric tumor cells [122]. The role of stromal cell-derived exosomes as mediators of tumor cell tumorigenicity was also described in a breast cancer model using embryonal fibroblasts [123]. Notably, the bone marrow is a sanctuary niche for stemness maintenance, with enrichment in stromal cells and particularly MSCs with key regulatory functions, and was shown to regulate tumor cell dormancy through exosome transference [124].

Once tumor cells arrive in a secondary site, they will interact with cells in the microenvironment, thus strengthening the “soil”. One of these interactions may involve tumor cell-derived exosomes. Related to this, exosomes from melanoma cells were shown to promote pro-metastatic features in BM-derived progenitor cells, establishing a crosstalk via exosome transfer between BM-derived stem cells and tumor cells, that, in turn, would promote metastatic growth [125]. Likewise, in an ovarian cancer model, AT-MSCs could be activated into myofibroblasts through exosomal transference from tumor cells [126]. This is very interesting, since it suggests sequential events involving tumor cells that arrive into a secondary site, modify functional properties in surrounding cells, and this in turn would enhance prometastatic features in new entrant tumor cells. Concomitantly, this in turn would recruit BM-MSCs or other distant MSCs that would acquire prometastatic features. This “circular” chain of events not only involves cell-cell contact and the secretion of molecules as paradigmatic mechanisms, but also cell vesicle-derived horizontal transfer of non-random packed molecules between cells. This concept deepens the scope of influence of cells residing in distant sites, since they should not necessarily leave a primary site to influence a secondary site.

**Figure 1:**
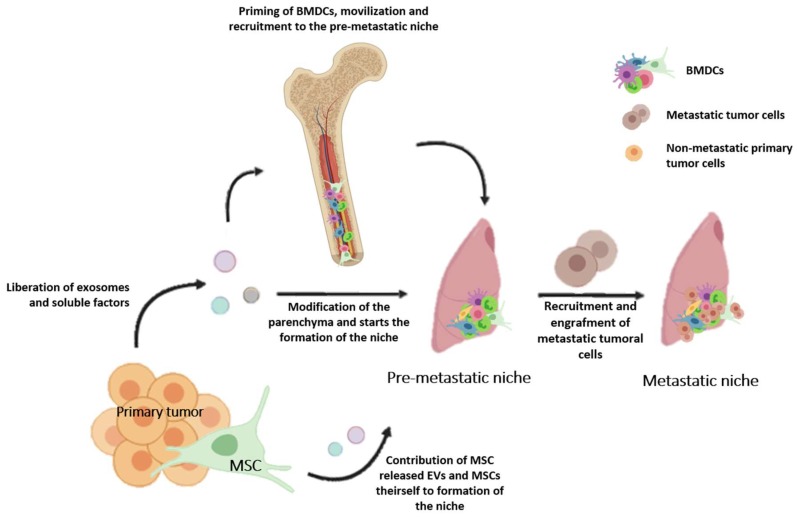
Representation of the steps necessary for the formation of the lung metastatic niche. Extracellular vesicles (EVs) and exosomes released by the primary tumor can directly modify the lung parenchyma or indirectly prime bone marrow derived cells (BMDCs) and recruit them into the lung pre-metastatic niche. MSCs also participates in this process by releasing EVs or migrating towards the inflammatory environment that the pre-metastatic niche represents. BMDCs (MSCs included) and enriched fibronectin environments may represent an attractive docking site for the disseminating tumor cells. Once metastatic tumoral cells are recruited and engrafted into the pre-metastatic niche, it allows for the formation of the lung metastatic niche.

A tumor that critically needs an understanding of the biological mechanisms underlying metastatic progression to improve its treatment is osteosarcoma (OS). OS is the most common bone malignant tumor, mainly affecting children and young adults [127]. Patients diagnosed with non-metastatic OS have a five-year survival rate close to 75%, while patients with pulmonary metastasis at diagnosis have 15%–30% survival rate [128]. MSCs have been implicated as possible cells of origin and also as a stromal component that, once recruited into the primary tumor, are “educated” by the tumor context and become relevant for the following reasons: (1) Once activated they adopt an immunosuppressive phenotype that inhibits proliferation of T, NK, and dendritic cells; (2) they interact with OS cancer stem cells and promote their proliferation, stemness, and migration by the release of IL-6 [129,130]; (3) they mediate the chemoresistance phenotype by the aberrant activation of STAT3 pathways in tumor cells [117,131]; (4) they favor migration and metastatic progression into the lungs [97]; (5) they induce a swift in-tumor metabolic phenotype, also known as the Warburg effect [132] (Figure 1).

Even though this revision focused on MSCs and exosomes and their role in the pre-metastatic niche establishment and metastatic progression, a determinant ability to ensure the biological success of cancer cells is the acquisition of chemoresistance mechanisms [133]. In the last years, exosomes have been implicated in the acquisition of chemoresistance in sensitive cells by the horizontal transmission of miRNA (miR-100, miR-222, miR-30a and miR-155) and proteins (ephrin type-A receptor 2, ABCG2) from resistant to sensitive cells, allowing the second ones to adopt a resistant phenotype [134,135,136,137]. Another important role of exosomes in chemoresistance is the possibility to represent a mechanism to sequester chemotherapeutic agents, contributing to diminishing the intracellular concentration of drugs [138,139]. All this points to the importance of understanding what is behind exosome signaling and how this signaling could be modulated in pursuing clinical advantages.

## 6. Therapeutic Potential of Exosomes

The possibility of utilizing a cell product that can be collected, instead of using the whole cell, brings advantages when considering possible therapeutic applications. As with any type of cell that has suffered an ex vivo expansion, there are risks associated with strategies that involve culturing MSCs in order to obtain large numbers of cells suitable for treatment [140]. Malignant transformation of a cell manipulated outside of its niche is an ever-present concern. In this context, strategies that involve the use of a low number of cells, or in the case of AT-MSCs, the alternative use of the stromal vascular fraction (SVF) are trustworthy substitutes and refer to a smaller than usual number of cells needed, since the percentage of MSCs present in the SVF, although higher than in BM, is still low [141]. These also point at the current paradigm that the biological action of MSCs would be exerted by EVs as their main effectors. According to this, it makes sense that a small dose of MSCs, as that contained in the SVF, could account for a therapeutic effect.

Several studies have demonstrated that exosomes can contain specific information of the tissues and/or cell types that produce them and especially with genetic material carrying the somatic mutations of each tumor. This circulating information in fluids, known as liquid biopsy, if properly sequenced, would allow for the identification of the clonal strain of origin and the presence of mutations of sensitivity and/ or resistance to targeted drug treatments. This could become a powerful tool for non-invasive diagnostics, therapeutics, and follow-up for almost all tumors, including relapses, metastatic development, as well as minimal residual disease biomarkers. Furthermore, perhaps in the near future, specific MSCs-derived exosomes could to help to identify the environmental changes promoting the neoplastic transformations of different tissues [142,143].

The therapeutic action of MSCs has been demonstrated in multiple disease settings with a main role as regeneration-inducing cells, with plenty of research in the fields of cardiology and neurology, among others. Given that MSCs have a high tropism for tumors, a number of approaches have intended their use as trojan horses to deliver therapeutic molecules [4,36]. Furthermore, clinical trials were conducted to evaluate the efficacy of MSCs, both naïve or loaded with therapeutic genes [140]. It would be of great relevance to deeply evaluate the effects of the particulate fraction from MSCs in these settings. Of relevance, during OS progression, since MSCs would be determinant in the formation of the pre-metastatic niche, a profound knowledge on the content of the exosomes and EVs, and their effects on the arriving metastatic cells, would allow for a better knowledge of upregulated molecules with the value of prognostic biomarkers, as well as future therapeutic approaches that would permit the manipulation of the stroma.

## 7. Conclusion Remarks

From the complex picture that emerges from the interaction between tumor cells and their environment, exosomes appear as new actors to add to this complexity. The canonical signaling pathways paradigm has dictated that soluble molecules, released by any cell type, could achieve functional responses and modulate a niche. The presence of a given cell in a new niche was almost determinant and critical for the niche establishment. However, the emergence of exosomes with non-random cargo, and their relationship with the cell that originated them, opens a new paradigm and broadens our understanding of the biological intricacy that underlies all microenvironmental signaling within a tissue. In this way, a tumor as an inflammatory remodeling and pathological tissue could affect the host in many more ways than expected, given that a primary tumor may precondition a future secondary growth site, not necessarily with migrating cells but with exosomes from non-migrating cells. Furthermore, all cell populations within a tumor (primary or metastatic) could communicate via this so-called communicasome. Adding to the scenario, exosomes uncover many possibilities of being used as specific microparticles, able to reach distant sites in a selective way, depicting them as promising and useful tools for diagnosis as well as for the design of new therapeutic strategies.

## Abbreviations

AT-MSCs	adipose tissue derived mesenchymal stem cells
BM	bone marrow
BMDCs	bone marrow derived cells
BM-MSCs	bone marrow derived mesenchymal stem cells
CD	cluster of differentiation
CXCR	CXC chemokine receptors
CCR	CC chemokine receptors
EC	endothelial cell
EMT	epithelial to mesenchymal transition
EVs	extracellular vesicles
GF	growth factor
IL-1β	interleukin 1-beta
ISEV	international society for extracellular vesicles
ITGs	integrins
MET	mesenchymal to epithelial transition
miR	microRNA
MMPs	metalloproteinases
MSCs	mesenchymal stem cells
MVB	multivesicular body
NK	natural killers
OS	osteosarcoma
PD-L1	programmed death ligand 1
PD-1	programmed death receptor 1
PGF/PlGF	placental growth factor
PM	plasmatic membrane
TAMs	tumor associated macrophages
TAN	tumor associated neutrophils
TGF-β	transforming growth factor- beta
TME	tumor microenvironment
TNF-α	tumor necrosis factor alpha
Treg	T regulatory cells
SVF	stromal vascular fraction
VEGFR-1	vascular endothelial growth factor receptor 1
